# Profile of subjective quality of life and its correlates in a nation-wide sample of high school students in an Arab setting using the WHOQOL-Bref

**DOI:** 10.1186/1471-244X-11-71

**Published:** 2011-04-25

**Authors:** Ghenaim A Al-Fayez, Jude U Ohaeri

**Affiliations:** 1 Department of Psychiatry, Faculty of Medicine, Kuwait University, Kuwait; 2 Department of Psychiatry, Psychological Medicine Hospital, Gamal Abdul Naser Road, P.O. Box 4081, Safat, Kuwait

**Keywords:** Quality of life, students, Arab, gender, age, parents

## Abstract

**Background:**

The upsurge of interest in the quality of life (QOL) of children is in line with the 1989 Convention on the Rights of the Child, which stressed the child's right to adequate circumstances for physical, mental, and social development. The study's objectives were to: (i) highlight how satisfied Kuwaiti high school students were with life circumstances as in the WHOQOL-Bref; (ii) assess the prevalence of at risk status for impaired QOL and establish the QOL domain normative values; and (iii) examine the relationship of QOL with personal, parental, and socio-environmental factors.

**Method:**

A nation-wide sample of students in senior classes in government high schools (N = 4467, 48.6% boys; aged 14-23 years) completed questionnaires that included the WHOQOL-Bref.

**Results:**

Using Cummins' norm of 70% - 80%, we found that, as a group, they barely achieved the well-being threshold score for physical health (70%), social relations (72.8%), environment (70.8%) and general facet (70.2%), but not for psychological health (61.9%). These scores were lower than those reported from other countries. Using the recommended cut-off of <1*SD *of population mean, the prevalence of at risk status for impaired QOL was 12.9% - 18.8% (population age-adjusted: 15.9% - 21.1%). In all domains, boys had significantly higher QOL than girls, mediated by anxiety/depression; while the younger ones had significantly higher QOL (*p *< 0.001), mediated by difficulty with studies and social relations. Although poorer QOL was significantly associated with parental divorce and father's low socio-economic status, the most important predictors of poorer QOL were perception of poor emotional relationship between the parents, poor self-esteem and difficulty with studies.

**Conclusion:**

Poorer QOL seemed to reflect a circumstance of social disadvantage and poor psychosocial well-being in which girls fared worse than boys. The findings indicate that programs that address parental harmony and school programs that promote study-friendly atmospheres could help to improve psychosocial well-being. The application of QOL as a school population health measure may facilitate risk assessment and the tracking of health status.

## Background

The upsurge of interest in the quality of life (QOL) of children in general population samples is in line with the 1989 Convention on the Rights of the Child, which stressed the child's right to adequate circumstances of physical, mental, and social development [1,2]. While most of the general population studies have emanated from the western world [3-11], a few have come from Asia [12-14], South America [15] and Iran [16]. There are no such reports from the Arab world. The lone report on QOL for students from an Arab country was based on a convenience sample of 224 college students and was focused on the relationship with intensity of religiosity [17].

Although various authors have recommended that the assessment of QOL among adolescents should include contextual variables that are not generally regarded as health-related (e.g., satisfaction with family and peer relationships and family income) [5,18-23], most reports have been based on health-related quality of life (HRQOL) measures [reviewed, [1,24]]. Only a few have used instruments that attempt to cover the broader issues of QOL [7,11,13], including a modification of the WHO Quality of Life Instrument (WHOQOLI) [13,25]. QOL measures that focus on the construct of HRQOL have been criticized on the grounds that their narrow focus on the impact of health conditions on physical, psychological and social functioning implies that full health equates to maximum QOL [24,26-29]. In a critique of six definitions of QOL, it was suggested that defining QOL in terms of life satisfaction is the most appropriate [29]. Instruments for pediatric QOL assessment should have conceptually strong underpinnings[24].

It is important to assess the QOL of adolescents and young adults of school age using instruments that include contextual variables, because the vast majority are healthy [30]; and since QOL is sensitive to distress in various domains of living [31], the data can help to provide information beyond symptoms, to identify an otherwise undetectable high risk group for problems [32]. For such a population, reliance on the traditional measures of health could lead to under-identification of psychosocial problems, "the new hidden morbidity" [5,33]. In view of the above considerations, we have used the short version of the WHOQOLI (the WHOQOL-Bref) to assess the subjective QOL of a nation - wide sample of senior high school students; first, because the items emphasize satisfaction with life circumstances [24,29], and the domains encompass health-related and contextual issues that have been found to be important for adolescents [19,34]. Second, the Arabic translation of the WHOQOL-Bref has been shown to have satisfactory reliability and validity indices in general population and clinical samples [35,36]. Third, the WHOQOL-Bref was simultaneously developed in diverse cultures, thus overcoming the usual controversy over the problem of applying a questionnaire articulated in one culture in a different culture [25,37]. It is noteworthy that the original validation sample for the WHOQOL-Bref [25] included adolescents (aged 12 - 19 yrs). In other words, the WHOQOL-Bref is judged to be appropriate for the age group that we studied. We have done the assessment using the model described by Jirojanakul et al [13]. In this model, personal and parental background factors, general health factors and socio-environment factors are all significantly associated with QOL.

This is based on the evidence that personal background factors have been found to be associated with QOL. Thus, while reports of adolescent samples (>12 years) consistently found that poorer QOL was significantly associated with female gender and older age [3,5,16,38-41], the reports that involved children who were less than 12 years of age either found that there were no significant gender differences [42], or that the girls had significantly better QOL [13,14,43]. In addition, poorer QOL was associated with poor physical health, psychic distress, and low self-esteem [7-9]. Of the parental factors, the consistent findings are that, poorer QOL was associated with parental low socio-economic status, low educational attainment, and divorce [3-5,7-10,15,44,45]. Of the socio-environmental factors, parental stress and the quality of emotional relationship between the parents were found to have long-term implications for the child's well-being [6,9,46,47]. Interestingly, children can reliably report on the quality of emotional relationship between their parents, while parents can predict children's response about parental relationship [46,48]. Furthermore, better QOL was significantly associated with easy access to health service, lack of feeling of difficulty at school, and connectedness with school [5,8,49]. It has been suggested that older adolescents tend to have poorer QOL, possibly because they are exposed to greater social demands and stresses, such as increased academic, emotional and other social pressures, so that they tend to have relatively more difficult life situations to contend with, in comparison with the younger ones [19].

At the conceptual level, a notable problem with QOL data is the interpretation of what the data mean. This problem concerns the issues of a cut-off score for poorer QOL or the identification of subjects "at risk status for impaired QOL" [30], and the clinical significance of the scores [50,51]. An important helpful step in this regard is the use of scales whose domains are aggregated into percentage maximum score of 0 to 100 (i.e., % scale maximum or % SM method). In a review of several studies from the western world, it was found that the average score for healthy populations tended to be in the range of 70 - 80% SM [22,52]. Accordingly, it was suggested that subjective well-being could be operating within a psychological homeostatic regulation system (like body temperature) that is represented by a score of 70%-80% of scale maximum for QOL instruments [22]. It appears that this recommendation is relevant for pediatric populations, too. For example, in a review of the Pediatric Quality of Life (PedsQOL) [4] domain scores of six studies in large samples of school children from the western world, it was shown that in five of them, the average scores for the domains of QOL (using child ratings) ranged from 72.9% to 91.1% [9]. In the sixth study, the children had a mean total PedsQOL score of 67.2, which the authors considered to be relatively low [8]. Other studies from Finland (75.4% - 85.0%) [38] and the USA (78.2% - 84.0%) [30] had similar findings. This is supported by similar data from non-western countries, such as Korea (82.6% - 93.5%) [12], Brazil (73.0% - 93.1%) [15], and Iran (71.7% - 90.9%) [16]. For another questionnaire, the Generic Children's Quality of Life Measure, a UK study reported 72.7% - 75.3% [11]. Using another yardstick, it has been suggested that a QOL domain score of 1 standard deviation (1*SD*) below the population mean would probably help to identify subjects at risk for impaired QOL [9,30,53], because such scores represent scale scores similar to those of children with severe chronic health conditions [30].

The design of our study was guided by the issues highlighted above. With regard to those issues, the Kuwaiti perspective is important because it adds the contribution from a country where, for nationals, there is an effective national social welfare system, health care services are free and easily accessible; and the conservative Muslim culture, with traditional gender roles and sexual segregation, prevails. It has been suggested that QOL is context-specific [13].

### Objectives

The specific objectives of the study were to:

(i) highlight how satisfied Kuwaiti senior high school students were with life circumstances as in the WHOQOL-Bref; (ii) estimate the prevalence of at risk status for impaired QOL, and establish the QOL domain score normative values, in comparison with the international data [25]; (iii) examine the relationship of QOL with personal factors (socio-demographic variables), general health factors (subjects' perception of being currently ill, and their scores on scales for anxiety, depression and self-esteem); parental factors (parental employment, educational and marital status); and socio-environment factors (perceived difficulty with studies and social relationships, and perceived quality of emotional relationship between the parents).

We hypothesized that, in view of the widely noted importance of parental material well-being and health access:

- Kuwaiti students would be generally satisfied with their circumstances of living,

- and their average QOL domain scores would be high, in comparison with the international data.

- In view of the robust findings in the literature, however, poorer QOL would be significantly associated with female gender, older age, high scores on anxiety and depression, low self-esteem and poor perception of the emotional relationship between the parents.

## Methods

### Participants and setting

Kuwait is an oil - rich Arab country, located in the Arabian Gulf. Of the total 3.4 million population, Kuwaiti nationals make up about 1.1 million (48.9% male, 51.1% female) (2007 census). There are six administrative districts or governorates. About 97% live in urban areas, and the unemployment rate is 2.3% (2004 estimate). According to the 2007 census data from the Kuwait Public Authority for Civil Information (PACI), those aged 14 - 23 years (our sample age range) (212144) constituted 20.4% of nationals (50.5% male, 49.5% female). Our sample size was guided by the recommendation of the International Quality of Life Assessment (IQOLA) project researchers, that the sample size for general population norming should be 2500 - 3000 [54]. This would allow for comparison of scale scores by gender and 10 - year age groups.

The study took place in Kuwaiti government secondary schools in all the governorates. All such schools are sexually segregated. Accordingly, the sampling strategy was aimed at representing the three types of schools, viz: boys', girls', and the credit-hour system (i.e., senior high schools where students have the option to choose three subjects per session). The focus was on students in the senior classes, consisting of grades (class years) 10, 11, and 12. This is because the questionnaires are self - rated and there was need to focus on an age group that would not have difficulty understanding and completing them. In 2006/7, a nationwide sample of 4467 senior high school students (mean age 16.9, *SD *= 1.2 yrs, range = 14 - 23) in Kuwaiti government secondary schools was studied, with adequate representation of the governorates and gender (48.6% boys). The participants hailed predominantly from fairly large, stable and harmonious family homes (83.1% rated parental relationship as good/excellent; 85.1% of parents lived together, and average sibling size was 6.3). Most fathers (73.3%) were gainfully employed. Of the 4442 (99.4%) who stated their nationality, 3771 (87.3%) were Kuwaitis, 69 (1.6%) were stateless citizens ("bedoons"), and 458 (10.3%) were from other Arab countries, especially the Arabian Gulf states.

### Procedure

First, a list of all the government secondary schools was obtained from the Ministry of Education. Six schools were randomly selected from each of the six governorates (total, 36 schools), viz: two each from boys', girls' and credit-hour system. From each selected school, two classes each from grades 10 and 11, and one class from grade 12 were randomly selected, in order to proportionately represent the number of classes in each grade.

### Ethical considerations

The study was carried out in compliance with the Helsinki Declaration. Hence, the protocol for all aspects of the study, including the pilot testing of the questionnaires, was approved by the institutional review boards of the Kuwait Ministry of Education and the Kuwait Society for the Advancement of Arab Children (KSAAC). Thereafter, the Principal of each selected school was approached for approval and for the cooperation of the school's psychologists. At the preliminary stage of the study, the research team hosted the psychologists of the selected schools at meetings facilitated by the Ministry of Education.

A few days after explaining the objectives of the study to the selected classes, the schools' psychologists and class prefects chose dates and times convenient to the study schedule. In the few days between explaining the nature of the study and the completion of the questionnaires, the students were requested to inform their parents about the study, in case any parents would refuse. It was emphasized that refusal to participate would not lead to any form of punishment. In the Kuwaiti culture, this method of obtaining informed consent from the Ministry of Education, the KSAAC, and the school principals, is deemed sufficiently ethical for such a study. Moreover, the questionnaires were completed in class, under the supervision of school psychologists whom the students and their parents were familiar with. There were no refusals by parents and students. In order to ensure adequate supervision and explain possibly difficult items, the school psychologists stayed with them in class while the students completed the questionnaires, anonymously. All students in the selected classes agreed to complete the questionnaires. Although we did not record the number of students who were not present in school for the selected classes on the days of the study, our impression was that this number was probably very small and not obvious to the school psychologists.

### Pilot testing of the questionnaires

Before the commencement of the study, the questionnaires were translated into Arabic by the method of back - translation. The research team critically examined the instruments and presented them to senior mental health workers to examine the face validity of the contents. Thereafter, the modified version, as detailed below, was pilot tested among students (50 boys and 50 girls), from two schools that were not part of the main study, using the same methodology as described above. Test - retest reliability was assessed by analyzing the responses of 55 subjects (from the 100) who volunteered to complete the final questionnaires twice in a four - week period.

### Operational definitions

We accepted the WHO definition of QOL as individuals' perception of life in the context of the culture and value system in which they live and in relation to their goals, expectations, standards and concerns [25]. This was the conceptual framework for articulating the WHOQOL Instrument [34]. It has also been adopted as the conceptual framework for a measure of QOL for children [55]. Our focus was on subjective QOL, as distinct from objective QOL [56].

We defined subjects' satisfaction as the level of positive appreciation for each item of the WHOQOL-Bref [29]. That is, we used the idea of satisfaction for an item as a rating of more than average or neutral point [57,58], which in the case of the WHOQOL-Bref varies (according to the wording of the item) as: good/very good; mostly/completely; or satisfied/very satisfied. Hence, we quantified the group's satisfaction with each item as at least 50% of respondents in the group rating the item as good/very good; dissatisfaction (< 50%); bare satisfaction (50 - 65%); moderate satisfaction (66 - 74%); and highest satisfaction (≥ 75%) [56].

### The WHOQOL - Bref

This is a 26 - item self - administered generic questionnaire, being a short version of the WHOQOL - 100 scale [25]. The response options range from1 (very dissatisfied/very poor) to 5 (very satisfied/very good). It emphasizes the subjective responses rather than objective life conditions, with assessment made over the preceding two weeks. It consists of domains (or dimensions) and a facet (or sub - domain). The items on "overall rating of QOL" (OQOL) and subjective satisfaction with health, are not included in the domains, but are used to constitute the general facet on OQOL and general health (general facet). The more popular model for interpreting the scores has four domains, namely, physical health (seven items), psychological health (six items), social relations (three items) and environment (eight items). Our analysis was based on this model.

The domain scores of the WHOQOL-Bref can be computed in three ways. The first is a summation of the raw scores of the constituent items. The second and third ways consist of transforming the raw scores. In the second way, the raw scores are transformed into scores that range from 4-20, to be in line with the WHOQL -100 Instrument. The third way, which is the percentage scale maximum (% SM) is a standardized conversion of Likert scale data projected onto a 0-100 scale. The WHOQOL Group has provided guidelines for these conversions [59]. The value of the later transformed score method (i.e., % SM) is that it can be used for making comparison with other scales [52].

There was need to modify the framing of some items of the WHOQOL-Bref in order to make them suitable to the circumstances of school age persons in this culture. First, the WHOQOL has no item on "school". Second, high school students in this culture are entirely dependent on their parents for financial and transportation needs. Third, by law, they are prohibited from engaging in romantic sexual activities. Accordingly, following the methods in the literature [1,23], we modified the following items of the WHOQOL-Bref to read thus:

(a) Item 12, on money: " How satisfied are you with the money available in your family for your care"; (b) Item 18: "How satisfied are you with your ability to do your school work"; (c) Item 21: "How satisfied are you with your sexual feelings"; (d) Item 24: "How satisfied are you with access to health services"; (e) Item 25: "How satisfied are you with the transportation facilities available to you."

In order to determine whether the pattern of response to the five modified items differed from the pattern of response to the other items, we examined the floor effects (i.e. % of subjects who rated themselves as "very dissatisfied" with each item) and ceiling effects (i.e. % of subjects who rated themselves as "very satisfied" with each item) for the five items, in comparison with those of the other items, and the WHO validating data [25]. Using the data for all participants (N = 4467), we found that the floor effect for the five modified items (2.2% - 8.7%) was similar to the range for the other items (1.6% - 8.1%), and the WHO data (1.7% - 8.8%). Also, the ceiling effect for the five items (17.6% - 53.9%) was within the range for the other items (13.1% - 59.2%), and comparable with the WHO data (10.1% - 35.2%).

Test - retest reliability (intra class correlation coefficient) for 39 subjects with full data for the retest exercise at the preliminary stage of the study was 0.95(95% C. I. = 0.92 - 0.97). For the entire population of participants (N = 4467), the alpha coefficient (internal consistency) for the WHOQOL-Bref was 0.91.

QOL domain scores (range 0 - 100%) were generated by organizing the items into the four domains as recommended by the WHOQOL study group [59]. Thereafter, we computed values for the domains corresponding with the 14-15; 16-17; 18 - 19; and 20-23 - year age groups. To determine the prevalence of those at risk status for impaired QOL, we dichotomized the domain scores at <*1SD *of the population mean [30]. Based on our results, the cut - off scores were <53.7 (physical health), < 44.1 (psychological health), <50.8 (social relations), <52.4 (environment domain), and <47.2 (general facet on health & OQOL).. Using the national census data, the prevalence rate of at risk status for poor QOL in each domain was adjusted by age and sex to the Kuwaiti population, in order to estimate the number of people with poor QOL at the ages we studied in the national population.

### Psychological distress and self-esteem

Designated items for anxiety, depression and anger were selected from the Trauma Symptom Checklist for Children, by Briere [60]. This was because our methodology could not be used to diagnose anxiety and depression, and we wished to reduce respondent burden and ensure reliability of responses [22,23]. The following items were chosen because they were most reflective of the corresponding American DSM-IV^TR ^symptoms: (a) Anxiety: Items 2, 15, 32, and 41; (b) Depression: Items 7, 9, 28, 42, and 52. Item 52 was modified because of the sanctions by the Islamic culture on suicide, to read: "Wishing I were dead"; (c) Anger: Items 19, 16, 21 and 22.

Test - retest reliability (intra class correlation coefficient) for 47 subjects with full data for the retest exercise at the preliminary stage of the study was 0.90(95% C. I. = 0.85 - 0.94). For the entire population of participants (*N *= 4467), internal consistency was 0.87. The item scores were summed up to generate total scores for anxiety and depression.

The 10-item Rosenberg's scale [61] was used to assess self-esteem.

### Socio-environmental factors

As there are no available formal instruments to assess the socio-environmental factors of interest to this study, and in order to reduce respondent burden [23], we assessed this domain with a few number of items that we articulated specifically for this study, based on our clinical experience of children in this culture, thus:

(i) one item on perceived quality of emotional relationship between the parents (response options: poor, fair, good, excellent) [6,9]; (ii) difficulty in psychosocial functioning. This was assessed by three items concerning difficulties being encountered as a result of various activities, viz: difficulty with studies (yes/no); difficulty relating with friends (yes/no); experiencing any other difficulties (yes/no); (iii) one item on perceived need for medical or psychological help (response options: no problem, need help only from friends, need medical/psychological help but not receiving it, need medical/psychological help and receiving it).

### Data analysis

Data were analyzed by SPSS version 15 for Windows (SPSS Inc., Chicago, Illinois). We examined the frequency distribution of the scores. Since the QOL domains scores were not normally distributed, we examined the association of socio-demographic factors and self - rated current illness with QOL domain scores using non - parametric tests of significance (Kruskal - Walis' chi-square and Mann-Whitney U test); and used Spearman's correlation to assess the relationship between anxiety/depression scores and QOL domain scores. The possible contribution of covariates (e.g., anxiety, depression, self-esteem and psychosocial difficulties) to sex and age differences in QOL scores was assessed by analysis of covariance (ANCOVA). We used multiple regression analyses to assess the associations of QOL in the multivariate context, with scores on the general facet and each of the domains as dependent variables. Based on the literature [31], the independent variables were entered in five different blocks, thus: Step 1: background socio-demographics; step 2: the quality of parental emotional relationship, difficulty with studies and difficulty with social relationships; step 3: self-esteem score; step 4: anxiety score; and step 5: depression score. Multi-collinearity was assessed by the values of "tolerance" (cut-off score </= 0.2) and variance inflation factor (VIF - cut-off score >4.0) [62]. The level of statistical significance was set at *p *< 0.05. Missing data were handled by excluding cases analysis by analysis.

## Results

### Satisfaction with circumstances of life: (Table 1)

**Table 1:** Frequency distribution of WHOQOL-Bref items*

Highly satisfied**:		Moderate satisfaction**:		Bare satisfaction**:		Dissatisfied**:	
Item	%	Item	%	Item	%	Item%	%
Ability to get around	85.3	Self-satisfaction	68.2	Overall QOL	63.9	Ability to concentrate	40.7
Condition of place of living	74.9	Transport	73.7	No physical pain	60.8	Leisure opportunity	45.6
Need for treatment	74.5	Health	65.8	Enjoyment of life	50.6	No negative feeling	18.4
Money	76.7	Safety	70.2	Physical environment	54.8		
		Personal relations	72.2	Bodily appearance	63.5		
		Energy	72.5	Available information for health	54.6		
		Friends' support	67.8	Activities of daily living	57.5		
		Access health services	67.2	Sexual feeling(63.6);	63.6		
				Life meaningful(57.4);	57.4		
				Sleep	55.4		

* Because of missing data, N was variously: 4407 - 4458

** Operational definition: We quantified satisfaction with each item as at least 50% of respondents in the sample positively appreciating the item (i.e., proportion of subjects in the group who rated satisfaction for the item as "satisfied" or "very satisfied"); dissatisfaction (<50% were satisfied/very satisfied with item); bare satisfaction (50 - 65%); moderate satisfaction (66 - 74%); and highly satisfied (≥ 75%)[51,56]

Using the criteria previously defined, we found that the pattern of satisfaction was in line with their material circumstance. Hence, for a mostly healthy population in a materially affluent and conservative society, at least three - quarters of subjects felt satisfied with availability of money for their needs (3382/4407 or 76.7%), and felt no need for treatment (3286/4409 or 74.5%); while over two-thirds were satisfied with access to health services (2969/4421 or 67.2%). Furthermore, in line with the obvious restrictions on leisure opportunities in this society, less than one - half were satisfied with available opportunity for leisure activities (2014/4420 or 45.6%); and probably as indicative of their concern with their studies, they were also generally not satisfied with their ability to concentrate (1805/4431 or 40.7%).

### Pattern of QOL domain scores (Table 2 and Table 3)

**Table 2:** Normative values of subjective quality of life domain scores by age groups*.

	Physical health	Psychological health	Social relations Domain	Environment domain	General facet on health & OQOL
Age groups*:yrs: Males**	Mean(SD) [95% C.I]	Mean(SD) [95% C.I]	Mean(SD) [95% C.I]	Mean(SD) [95% C.I]	Mean(SD) [95% C.I]
14-15: N = 230	75.3(16.3) [73.1-77.4]	66.8(17.5) [64.5-69.2]	75.8(21) [73.0-78.5]	74.9(17.5) [72.6-77.3]	73.8(24) [70.7-76.9]
16-17: N = 1338	72.5(15.6) [71.6-73.3]	65.4(17.2) [64.2-66.3]	74.4(22) [73.3-75.6]	73.5(17.1) [72.0-74.5]	71.8(22) [71.6-73.9]
18-19: N = 527	69.0(15.5) [67.7-70.4]	64.4(17.1) [62.9-65.9]	73.6(22) [71.7-75.4]	70.5(17.6) [68.9-72.1]	69.9(23) [68.0-71.9]
20-23: N = 62	67.3(13.3) [63.8-70.8]	59.9(16.8) [55.6-64.3]	70.4(20) [65.2-75.6]	67.0(17.9) [62.3-71.7]	65.5(22) [60.0-71.0]
Total: N = 2157	71.8(15.7) [71.1-72.5]	65.1(17.2) [64.4-65.9]	74.2(21) [73.3-75.2]	72.8(17.4) [71.9-73.5]	72.0(23) [71.0-72.9]
Adjusted scores (SE): All males***	71.1(0.34) [70.4 - 71.7]	63.7(0.33) [63.1 - 64.4]	73.5(0.46) [72.6 - 74.4]	71.7(0.37) [70.9 - 72.4]	70.5(0.45) [69.6 - 71.4]
Females**	Physical health	Psychological health	Social relations	Environment domain	General facet on health & OQOL
14-15: N = 234	73.2(15.1) [71.2-75.2]	61.1(18.0) [58.8-63.5]	74.9(19) [72.3-77.4]	71.7(18.0) [69.3-74.1]	72.7(22) [69.8-75.5]
16-17: N = 1432	69.3(16.4) [68.4-70.1]	59.8(17.7) [58.9-60.7]	71.7(21) [70.6-72.9]	70.5(18.7) [69.5-71.5]	69.3(23) [68.1-70.5]
18-19: N = 554	64.6(16.8) [63.2-66.0]	56.7(17.6) [55.3-58.2]	69.3(22) [67.4-71.2]	64.9(19.6) [63.3-66.7]	65.7(24) [63.8-67.7]
20-23: N = 56	61.4(17.5) [56.4-66.3]	51.2(20.4) [45.7-56.7]	67.6(24) [61.1-74.2]	60.9(20.3) [55.3-66.5]	57.1(26) [50.2-64.1]
Total: N = 2276	68.4(16.6) [67.7-69.0]	58.9(17.0) [58.2-59.7]	71.3(21) [70.5-72.2]	69.0(19.1) [68.2-69.8]	68.5(23) [67.5-69.4]
Adjusted scores (SE): All females***	69.7(0.33) [69.1 - 70.4]	60.6(0.33) [59.9 - 61.3]	72.6(0.46) [71.7 - 73.5]	70.7(0.36) [69.9 - 71.4]	70.5(0.32) [69.7 - 71.4]
All participants: N = 4276	70.0(16.3) [69.5-70.5]	61.9(17.8) [61.4-62.5]	72.8(21) [72.1-73.4]	70.8(18.4) [70.3-71.4]	70.2(23) [69.5-70.9]
Adjusted scores (SE): All participants***	70.4(0.24) [69.9 - 70.9]	62.2(0.23) [61.7 - 62.6]	73.1(0.32) [72.4 - 73.7]	71.2(0.26) [70.7 - 71.7]	70.5(0.32) [69.9 - 71.1]

* Using the 0-100% scoring method: Mean (SD) [95% Confidence Intervals]

* In all domains and for both sexes, quality of life decreased with age, such that those aged 14-15 and 16-17 had significantly higher scores than those aged 18-19 and 20-23: KWχ^2 ^= 13.9 - 93.4, df = 3, *P *< 0.001..

** In all domains, males had significantly higher QOL than females: Mann-Whitney *U *= 1859917 - 2262080, *Z *= 5.2 - 11.6, *P *< 0.0001.

*** Adjusted for age, father's occupation, depression and anxiety scores.

Table 3 shows that the unadjusted prevalence of "at risk status for impaired QOL" [9,30] for various domains was 12.9% - 18.8%, while the age and sex adjusted rates were 15.9% - 21.1%.

**Table 3:** Prevalence of normal/poor (at risk status for impaired) QOL by gender*

	All study participants			Boys' QOL			Girls' QOL			Statistics
QOL Domains	Normal %	Poor %	Age adjusted **[95% C.I.]	Normal %	Poor %	Age adjusted **[95% C.I.]	Normal %	Poor %	Age adjusted** [95% C.I.]	*P *level Boys vs. Girls
Physical health N = 4276; Boys 2073 Girls 2203	82.1	17.9	21.1 [20.9-21.3]	85.4	14.6	15.9 [15.7 - 16.1]	79.0	21.0	27.1 [26.8-27.4]	0.0001
Psycholo-gical N = 4322 Boys 2091	84.2	15.8	19.2 [19.0 - 19.4	88.1	11.9	12.9 [12.7 - 13.1]	80.5	19.5	25.5 [25.2-25.8]	0.0001
Social relations N = 4273 Boys 2091	81.2	18.8	20.1 [19.9 - 20.3]	82.5	17.5	17.8 [17.6 - 18.0]	80.0	20.0	23.7 [23.4-23.9]	0.035
Environ-ment N = 4223 Boys 2031	84.3	15.7	18.7 [18.5 - 18.9]	87.7	12.3	15.7 [15.5 - 15.9]	81.2	18.8	21.5 [21.3-21.8]	0.0001
General facet N = 4456 Boys 2164	87.1	12.9	15.9 [15.8 - 16.1]	88.8	11.2	13.4 [13.2 - 13.6]	85.6	14.4	18.2 [17.9-18.4]	0.0001

* As defined by scores < 1*SD *population mean (see last row of Table 2 by gender) for each domain

** Prevalence rates were adjusted to the 2007 Kuwaiti population to estimate the number of children with at risk status for poor QOL at the ages that were studied in the general population

Using Cummins' recommendation of 70% - 80% [52], we found that, as a group, the students barely achieved the psychosocial well-being threshold score of 70% for all domains, except psychological health where they scored 61.9% (see Table 2, bottom rows). In particular, this pattern was characteristic of the boys aged 14 - 15 and 16 - 17. In the case of the girls, only the youngest achieved the threshold score of 70% for physical health, social relations and environment domains and general facet.

### Age and gender differences in QOL

In all domains and for both sexes, quality of life decreased with age, such that those aged 14-15 and 16-17 years had significantly higher scores than those aged 18-19 and 20-23 years (*KW*χ^2 ^= 13.9 - 93.4, df = 3, *p *< 0.001) (Table 2). Accordingly, in all domains, correlation of age with QOL was negative, though of small magnitude (*rho *= -0.07 - 0.16), but significant (*p *< 0.001).

In all domains, males had significantly higher QOL than females (*M-WU *= 1859917 - 2262080, Z = *5.2 *- 11.6, *p *< 0.0001), and there was a significantly higher prevalence of at risk status for impaired QOL among the girls (χ^2 ^ranged from 10.6 to 47.8, *df *= 1, *p *< 0.001 for all domains, except for social relations - χ^2 ^= 4.5- where the level of significance was *p *< 0.035) (Table 3).

### Other factors associated with QOL

There was consistent evidence of significantly poorer QOL with social disadvantage. Thus, in all domains, QOL was poorer for subjects whose parents were either divorced or fathers had either lower occupational status or were unemployed (*M-WU: *401233 - 475588, *Z *= 3.3 - 6.7, *p *< 0.001). For mother's occupational status, the trend reached significance only in the physical health domain (*KW*χ^2 ^= 7.5, *df *= 2, *p *< 0.02).

In all domains, QOL was significantly correlated with self-esteem (*rho *> 0.40), and negatively correlated with depression (*rho *> - 0.40). Also, domains of QOL were negatively correlated with anxiety (*rho *> - 0.40, *p *< 0.0001), except social relations (rho = - 0.29, *p *> 0.05). In all domains, QOL decreased with poorer perception of the quality of emotional relationship between parents (*KW*χ^2 ^= 315 - 767.9, *df *= 3, *p *< 0.0001). In all domains, those who expressed difficulty in social relationships, as well as with their studies, and general situations, and admitted having general health or psychological problems, had significantly poorer QOL (*M-W U *= 968408 - 1128935; *Z *= 15.1 - 21.0, *p *< 0.0001).

### Covariance analysis

It was necessary to do analysis of covariance in order to understand the impact of anxiety, depression, self-esteem and difficulty with studies and social relations on the noted age and gender differences in QOL. This is because these variables had gender and age differences, while being significantly associated with QOL. For example, the boys had significantly higher self-esteem scores than the girls (boys:30.7, SD 4.5, vs. girls: 30.1, SD 4.7) (*t *= 4.0, *df *= 4195, *p *< 0.001), while the girls had significantly higher anxiety (girls:13.9, SD 3.9, vs. boys: 12.9, SD 3.8) and depression scores (11.5, SD 3.7, vs. 10.4, SD 3.4) than the boys (*t *= 9.7-13.9, *df *= 4231, *p *< 0.001). Similarly, difficulty with studies (χ^2 ^= 49.7, *df *= 3, *p *< 0.0001) and social relationships (χ ^2 ^= 5.9, *df *= 1, *p *< 0.02) increased significantly with age.

In ANCOVA, we found that, after controlling for difficulty with studies and social relations, the previously noted age group differences in QOL narrowed considerably, such that the following pattern emerged:

(i) For psychological health and social relations, the differences were no longer significant (*p *> 0.05).

(ii) Physical health: those aged 14-15 had significantly higher scores (*p *< 0.001); but the scores for those aged 16-17 years (70.5%) were no longer significantly different from the scores for those aged 18-19 (67.4%) and 20-23 years (67.4%) (*p *> 0.05). The same pattern was noted for the environment domain and general facet.

Similarly, after adjusting for the scores on anxiety and depression, gender differences in QOL domain scores narrowed considerably in most domains (from *p <*0.0001 for the unadjusted scores) to produce the following pattern for boys and girls, respectively: (i) physical health: 70.9% vs. 69.4% (*p *< 0.002); (ii) psychological health: 63.5% vs. 60.6% (*p *< 0.0001); (iii) for environment: 71.7% vs. 70.5% (*p *< 0.02); and (iv) the differences were no longer significant for social relations (73.3% vs. 72.8%) and the general facet (70.1% vs. 70.5%) (*p *> 0.05).

However, the significant gender differences in quality of life seemed not to have been affected by difficulty with studies and social relationships (*p *< 0.001).

In other words, the mediators for age differences in QOL were difficulty with studies and social relations, while the mediators for gender differences were anxiety and depression.

### Regression analyses: associations of QOL in multivariate contexts (Table 4)

**Table 4:** Factors associated with domains of QOL in multivariate context*

Dependent variable	Independent variables or Predictors	% Variance or *R *square**	*Standardized beta*	*P*: level of significance***
General facet health/QOL	Age	1.4	-0.06	0.001
	Parental marital status	1.2	0.04	0.02
	Gender	0.5	0.008	0.58
	Father's occupation	0.4	0.03	0.05
	Parental emotional relationship	15.8	0.25	0.001
	Difficulty with studies	7.3	-0.11	0.001
	Difficulty in social relationships	0.8	0.006	0.72
	Self-esteem score	7.9	0.20	0.001
	Anxiety score	3.5	-0.12	0.001
	Depression score	1.5	-0.19	0.001
Physical health	Age	3.0	-0.12	0.001
	Gender	0.8	0.04	0.02
	Father's occupation	0.5	0.03	0.11
	Parental marital status	0.4	0.02	0.19
	Difficulty with studies	12.3	-0.15	0.001
	Parental emotional relationship	3.7	0.11	0.001
	Difficulty in social relationships	0.8	-0.02	0.39
	Self-esteem score	8.8	0.26	0.001
	Anxiety score	2.5	-0.15	0.001
	Depression score	0.2	-0.07	0.007
Psychological health	Gender	2.8	0.09	0.001
	Age	1.0	-0.05	0.001
	Difficulty with studies	14.7	-0.09	0.001
	Parental emotional relationship	8.6	0.17	0.001
	Self-esteem	18.6	0.36	0.001
	Anxiety	2.7	-0.06	0.001
	Depression	2.6	-0.26	0.001
Social relations	Age	0.6	-0.04	0.01
	Parental emotional relationship	6.1	0.12	0.001
	Difficulty in social relations	3.8	-0.05	0.007
	Difficulty with studies	0.6	0.003	0.88
	Self-esteem	9.0	0.25	0.001
	Anxiety	0.5	0.03	0.19
	Depression	2.1	-0.23	0.001
Environment	Age	1.6	-0.07	0.001
	Parental emotional relationship	17.7	0.28	0.001
	Difficulty with studies	7.4	-0.10	0.001
	Self - esteem	8.7	0.23	0.001
	Anxiety	1.9	-0.05	0.01
	Depression	1.9	-0.22	0.001

* Final stepwise regression model

** Total % of variance explained: general facet = 40.3; physical health = 33.2; psychological health = 54.4%; social relations = 24.0%; environment = 43.6%

*** Values of "tolerance" (cut-off score </= 0.2) and variance inflation factor (VIF - cut-off score >4.0) indicate no significant multi-collinearity.

#### Summary of predictors of QOL from the perspective of the conceptual framework

Using the model of Jirojanakul et al [13], the results of the regression analyses showed that variables from the personal factors (age and sex), parental factors (parental marital status and father's occupation), general health factors (self-esteem, anxiety and depression) and socio-environmental factors (quality of parental emotional relationship, difficulty with studies and social relationship) were variously important in predicting domains of QOL (Table 4). However, the variables that accounted for at least 5% of variance in any domain were: quality of parental emotional relationship (6.1% - 17.7%, except physical health, 3.7%), difficulty with studies (7.3% - 14.7%, except social relations, 0.6%), and self-esteem (7.9% - 18.6%). Although anxiety and depression contributed les than 4% of variance, they were consistently highly significant predictors (*p *< 0.001) of QOL, and played greater roles than the personal and parental background factors. In particular, the contribution of gender to various domains of QOL seemed to disappear when the psychological factors entered the equation. In other words, the contribution of personal and parental background factors to QOL seemed to be important because of the impact they had on the child's psychological status.

## Discussion

We assessed the subjective QOL of a nation-wide sample of Kuwaiti high school students using the WHOQOL-Bref, and examined the association of domains of QOL with several factors. This is the first of such a report from the Arab world for this age group. In line with the impression that QOL is sensitive to psychosocial distress [31], we found that the pattern of satisfaction was in consonance with the subjects' material and socio-cultural circumstances; and our findings indicated that poorer QOL was significantly associated with female gender, older age, indices of social disadvantage, psychic distress and social/academic pressures. What we have added to the literature are: the estimation of prevalence of at risk status for impaired QOL, thus making our findings clinically relevant as a population health outcome [4,7,9]; the presentation of normative values for this population (thus establishing benchmarks for comparison with clinical groups in the region); and the emphasis on the importance of child's perception of the quality of parental emotional relationship.

### Pattern of QOL domain scores

Although the average QOL scores of most domains for our subjects marginally met the 70% cut-off recommended by Cummins, and which is supported by data from several countries, the pattern of scores was similar to the international data because in all the available reports [9,11,12,15,16,30,38], the score for the psychological health domain was the least, in comparison with all other domains of QOL. It has been suggested that the low score on psychological health indicates that the students need access to programs and services that address their mental health needs [8]. The particularly low psychological health score for Kuwaiti students (61.9%) makes this recommendation highly relevant, especially for boys aged 20-23 years, and girls aged 16-23, who had average scores less than 60% (Table 2). This low score in the psychological health domain for our subjects is reflective of the reported relatively high rate of anxiety/depression morbidity among the youth in Kuwait (compared with the international data) [63-66].

Furthermore, judging by the average scores, it appears that the Kuwaiti students had lower QOL scores than their counterparts from other parts of the world. With regard to our finding of prevalence of at risk status for impaired QOL (12.9% - 18.8%), there are only data from Austria (15%) [9] and the USA (14-17%) [30] to compare with. Hence, there is need for more reports that present pediatric QOL data from the perspective of clinical relevance [51]. This perspective is important because it has been suggested that low QOL scores reflect children's perception of impaired psychological and physical health, with potential implications for the success of children in their living environments [8]. Hence, identifying the child with low QOL allows for early detection of hidden morbidity and health care needs [21]. In conclusion, our findings did not support our hypothesis that the average QOL domain scores for students in Kuwait would be high, in comparison with the international data. This dissonance between material living circumstance and QOL has been well noted in the literature [67,68].

### Factors associated with QOL

While our findings about gender and age differentials in QOL are similar to the international trend, the difference is that in Kuwait, the gender differences in QOL were more pronounced, affecting all domains at highly significant levels. The relatively low score in the psychological health domain (< 57%) for girls aged 18 - 23 exemplifies the situation for the older girls as has been described by Arab scholars, consequent on the socio-cultural situation [17,69,70]. What we have added to the literature is the finding that the gender differences in QOL scores were mediated by anxiety and depression. The implication is that the condition of the girls with problems can be alleviated by school health programs that focus on promotion of mental health. Similarly, our finding that the age differences in QOL were mediated by difficulties that the older students were experiencing with their greater burden of school work and demands for social relationships [19] implies that school - based programs that include making the school atmosphere more study - friendly have the potential to improve the QOL of students. These findings complement the results of our regression analyses. The finding about the predictive power of the child's perception of parental emotional relationship has been reported for psychopathology [71] and is supported by attachment theory [72]. The clinical implication is that those engaged in family work should emphasize the benefit of parental harmony on the well-being of the child [73].

Our finding on the role of the parental socio-economic situation supports the suggestion that children whose parents are socially disadvantaged need focused attention in school if their QOL is low [74].

### Limitations and strengths

The major limitation of the study is that it was cross-sectional; hence the results support an association, not causality. Moreover, the variables not measured, such as parental age, and monogamy/polygamy family setting could have contributed to the impact of quality of emotional relationship between the parents. The strengths of our study are that we studied a nation-wide sample using an internationally validated instrument, based on a conceptual framework, and we analyzed our data in such a way as to make QOL data clinically relevant as a population health measure. We needed to modify the item on sexual activity (because it is not appropriate in the culture) and it is arguable whether the replacement with sexual feeling is adequate. However, the adequate reliability indices of the instrument in our sample shows that the modifications we made have not diminished the noted satisfactory psychometric characteristics of the Arabic translation of the WHOQOL-Bref in this setting [36].

## Conclusion

The findings support the view that QOL is sensitive to psychosocial living situation. Hence, poor quality of life seemed to reflect a circumstance of social disadvantage and poor psychological well-being in which girls fared worse than boys. The findings indicate that programs that address parental harmony, as well school programs that promote student-friendly atmospheres (such as "School-Wide Positive Behavioral Interventions and Supports") [75] could help to improve the subjective QOL of the students.

## Competing interests

The authors declare that they have no competing interests.

## Authors' contributions

GAA and JUO designed the study, analyzed the data and prepared the manuscript. All the authors read the manuscript and approved it.

## Pre-publication history

The pre-publication history for this paper can be accessed here:

http://www.biomedcentral.com/1471-244X/11/71/prepub
